# Cysteine cathepsins B, X and K expression in peri-arteriolar glioblastoma stem cell niches

**DOI:** 10.1007/s10735-018-9787-y

**Published:** 2018-07-25

**Authors:** Barbara Breznik, Clara Limbaeck Stokin, Janko Kos, Mohammed Khurshed, Vashendriya V. V. Hira, Roman Bošnjak, Tamara T. Lah, Cornelis J. F. Van Noorden

**Affiliations:** 1 Department of Genetic Toxicology and Cancer Biology, National Institute of Biology, Večna Pot 111, 1000 Ljubljana, Slovenia; 2 International Postgraduate School Jozef Stefan, Jamova 39, 1000 Ljubljana, Slovenia; 3 Institute of Pathology, Faculty of Medicine, University of Ljubljana, Korytkova 2, 1000 Ljubljana, Slovenia; 4 Department of Pharmaceutical Biology, Faculty of Pharmacy, University of Ljubljana, 7 Aškerčeva cesta, 1000 Ljubljana, Slovenia; 5 Cancer Center Amsterdam, Department of Medical Biology at the Academic Medical Center, Amsterdam UMC, Meibergdreef 15, 1105 AZ Amsterdam, The Netherlands; 6 Department of Neurosurgery, University Clinical Centre Ljubljana, Zaloška cesta 7, 1000 Ljubljana, Slovenia

**Keywords:** Cysteine cathepsins, Glioblastoma stem cells, Niches, Stroma, Proteolytic activity

## Abstract

Glioblastoma (GBM) is the most lethal brain tumor also due to malignant and therapy-resistant GBM stem cells (GSCs) that are localized in protecting hypoxic GSC niches. Some members of the cysteine cathepsin family of proteases have been found to be upregulated in GBM. Cathepsin K gene expression is highly elevated in GBM tissue versus normal brain and it has been suggested to regulate GSC migration out of the niches. Here, we investigated the cellular distribution of cathepsins B, X and K in GBM tissue and whether these cathepsins are co-localized in GSC niches. Therefore, we determined expression of these cathepsins in serial paraffin sections of 14 human GBM samples and serial cryostat sections of two samples using immunohistochemistry and metabolic mapping of cathepsin activity using selective fluorogenic substrates. We detected cathepsins B, X and K in peri-arteriolar GSC niches in 9 out of 16 GBM samples, which were defined by co-expression of the GSC marker CD133, the niche marker stromal-derived factor-1α (SDF-1α) and smooth muscle actin as a marker for arterioles. The expression of cathepsin B and X was detected in stromal cells and cancer cells throughout the GBM sections, whereas cathepsin K expression was more restricted to arteriole-rich regions in the GBM sections. Metabolic mapping showed that cathepsin B, but not cathepsin K is active in GSC niches. On the basis of these findings, it is concluded that cathepsins B, X and K have distinct functions in GBM and that cathepsin K is the most likely GSC niche-related cathepsin of the three cathepsins investigated.

## Introduction

Glioblastoma (GBM) remains the most lethal and most frequently-occurring primary brain tumor (Louis et al. 2016). GBM is characterized by extensive cancer cell proliferation, vascularization, necrosis as well as invasion of GBM cells into surrounding brain parenchyma (Ohgaki and Kleinhues 2013; Cuddapah et al. 2014). Infiltration deep into normal brain far from primary tumor mass prevents complete surgical resection (Claes et al. 2007) and this has been found to be associated with expression and activity of proteolytic enzymes (Mentlein et al. 2012; Paw et al. 2015), such as lysosomal cysteine cathepsins (Lah et al. 2006), urokinase-type plasminogen activator (Gondi et al. 2004) and matrix metalloproteases (MMPs) (Li et al. 2016). Extracellular matrix (ECM) of GBM can be degraded by proteolytic activity to enable intracranial GBM cell trafficking.

A major cause of GBM recurrence is the presence of a highly tumorigenic and therapy-resistant subpopulation of GBM cells in tumors with characteristic expression of stem cell-related genes, the GBM stem cells (GSCs) (Lathia et al. 2015; Godlewski et al. 2017). GSC stemness is tightly regulated by the GSC microenvironment, specific micro-anatomical regions within the tumor, named GSC niches (Ross et al. 2017). Recently, Hira et al. (2015, 2018a, b) reported that GSCs are exclusively localized in hypoxic peri-arteriolar niches at the edge of the lamina adventitia of the arterioles. In these regions, maintenance and protection of GSCs is achieved by remodeling of their own microenvironment (Lathia et al. 2015) and by interactions with cells that are present in the niches (Hambardzumyan and Bergers 2015; Jackson et al. 2015).

Cysteine cathepsins, a group of lysosomal proteases belonging to the C1A family of papain-like proteases (https://www.ebi.ac.uk/merops/) (Kramer et al. 2017), have been shown to be associated with growth and invasive spread of GBM (Gondi et al. 2004; Claes et al. 2007; Gole et al. 2012; Breznik et al. 2017a). Cysteine cathepsins B, X and K are involved in various processes of cancer progression, including therapeutic resistance (Olson and Joyce 2015) and apoptosis (Kenig et al. 2011). On the other hand, certain cathepsins also play a role in tumor suppression (Lah et al. 2006; Lopez-Otin and Matrisian 2007). These seemingly conflicting roles depend on the tumor context, which emphasizes the importance of in vivo analysis to understand functions of cathepsins in the GBM micoenvironmental landscape (Quail and Joyce 2017).

Cathepsin B is recognized as the most pro-invasive lysosomal cathepsin compared to cathepsins L and S that did not affect GBM cell invasion in 3D spheroid models (Gole et al. 2012). The expression of cathepsin B is highly prognostic in various types of cancers, including GBM (Strojnik et al. 2005). Expression of cathepsin B correlates with brain tumor malignancy and it has been found to be an independent predictor of survival of GBM patients (Strojnik et al. 1999; Gondi et al. 2004; Colin et al. 2009). Cathepsin B activity in cancer progression was initially associated with pericellular initiation of proteolytic cascades (Gole et al. 2012; Olson and Joyce 2015) and with direct degradation of ECM components (Lah et al. 1989). Indirectly, cathepsin B affects invasive behavior of GBM cells by proteolytic processing of cytokines and growth factors, such as transforming growth factor β (TGF-β) (Breznik et al. 2017a; Mitrović et al. 2017). Noteworthy, cathepsin B was also implicated in the cross-talk between GBM cells and endothelial cells (Rempel et al. 2000; Calabrese et al. 2007), involving SDF-1α signaling (Kenig et al. 2010).

SDF-1α is present in high abundance in GSC niches and we have previously demonstrated that CXCR4/CXCR7-positive GSCs migrate towards a gradient of SDF-1α that retains GSCs in peri-arteriolar niches (Hira et al. 2017a). GSC niches seem to be similar to bone marrow hematopoietic stem cell (HSC) niches (Hira et al. 2018a). Staudt et al. (2012) observed that cathepins B, K, L and X are able to digest (although with different efficacy) SDF-1α, which is important for retaining HSCs in their niches. The authors reported that cysteine cathepsins B, K, L and X, constitutively secreted by osteoblasts, are part of the fine-tuned regulation of SDF-1α levels in the bone marrow. With respect to the SDF-1α patterns of proteolytic cleavages, cathepsins K and L are similar and therefore for this study cathepsins B, K and X were studied because of different mechanisms of digestion of this cytokine (Kenig et al. 2010).

We discovered recently that cathepin K among all protease genes was one of the five most highly differentially expressed in GBM tissues and cells vs. their normal counterparts (Verbovšek et al. 2014). Due to its osteolytic activity, cathepsin K has been mostly investigated in bone and cartilage tissue disorders and bone metastases. Cathepsin K belongs to the cathepsin L-like cluster of the C1A peptidase family. Its tetrameric form allows allosteric accommodation of negatively-charged glycosaminoglycans, enabling formation of complexes with unique collagenolytic activity (Novinec and Lenarčič 2013; Verbovšek et al. 2015), whereas the monomeric form of cathepsin K also degrades smaller proteins, including growth factors and cytokines, such as SDF-1α (Staudt et al. 2012). Being localized to specific niche areas in tumor tissues, we suggested its regulation of GSC migration out of the niches (Verbovšek et al. 2014; Hira et al. 2017a), in a similar way as reported by Kollet et al. (2012).

Increased cathepsin X expression and its strict exopeptidase activity have also been associated with various types of cancer (Vižin et al. 2014), but until now its expression in GBM has not been explored. Cathepsin X has been shown to promote cancer growth and invasion by compensation for cathepsin B proteolytic activity in the cathepsin B-deficient transgenic polyoma middle T oncogene (PymT)-induced breast cancer mouse model (Vasiljeva et al. 2006). Structure and activity of cathepsin X, formerly named cathepsin Z (Akkari et al. 2014), shows several unique features that distinguish it from other cysteine cathepsins (Kos et al. 2015). Initially, cathepsin X expression was found to be associated with cells of the immune system, regulating their proliferation, maturation, migration, adhesion, phagocytosis and signal transduction (Kos et al. 2009). Various molecular targets of cathepsin X exopeptidase activity were identified afterwards (Kos et al. 2015) and its expression has been detected in brain, localized in neurons, glial cells and ependymal cells (Wendt et al. 2007).

In the present study, we aimed to reveal the cellular distribution of the three cysteine cathepsins with related, though distinct specificities, in the regions of peri-arteriolar GSC niches.

## Materials and methods

### Patients and brain tumor samples

For paraffin-embedded tissue sections, GBM tumor biopsies were obtained from GBM patients that were operated at the Department of Neurosurgery, University Medical Centre of Ljubljana, Slovenia. The study was approved by the National Medical Ethics Committee of the Republic of Slovenia (approval no. 92/06/13). Altogether, 14 patients with GBM (glioma grade IV) were included. Tumor diagnoses were established by standard histopathology protocols at the Institute of Pathology of the Medical Faculty, University of Ljubljana. Briefly, tumors were classified according to the WHO classification 2007 (Louis et al. 2007) that was the most recent one at the time of diagnosis. Formalin-fixed, paraffin-embedded tissue was used for histological and immunohistochemical analyses. All cases were analyzed by immunohistochemistry for IDH1 mutation and when necessary for GFAP, Olig2, ATRX, and p53 expression. IDH1/2 status was confirmed by sequencing in a subset of cases. Clinical data of the patients are shown in Table 1.

**Table 1:** Clinical data of the 14 GBM patients

Number	Gender	Age at the time of operation (years)	Survival (months)	Newly-diagnosed or recurrent	Therapy (radio- or chemotherapy with temozolomide)	IDH1 mutation	Presence of GSC niches
1	M	46	17	Newly-diagnosed	Radiotherapy (60 Gy) + temozolomide, adjuvant temozolomide therapy	No	Yes
2	M	81	7	Newly-diagnosed	Radiotherapy (30 Gy)	No	No
3	F	62	32	Newly-diagnosed	Radiotherapy (60 Gy) + temozolomide, adjuvant temozolomide therapy	No	No
4	M	81	0	Newly-diagnosed	/	No	No
5	M	43	21	Newly-diagnosed	Radiotherapy (60 Gy) + temozolomide, adjuvant temozolomide therapy	Yes, R132H	No
6	M	54	3	Newly-diagnosed	Radiotherapy (60 Gy) + temozolomide, without adjuvant temozolomide therapy	No	Yes
7	M	55	26	Newly-diagnosed	Radiotherapy (60 Gy) + temozolomide, adjuvant temozolomide therapy	No	Yes
8	M	75	17	Newly-diagnosed	Radiotherapy (60 Gy) + temozolomide, adjuvant temozolomide therapy	No	No
9	M	66	8	Newly-diagnosed	Radiotherapy (60 Gy) + temozolomide, adjuvant temozolomide therapy	No	Yes
10	F	59	16	Newly-diagnosed	Radiotherapy (60 Gy) + temozolomide, without adjuvant temozolomide therapy	No	Yes
11	F	67	3	Newly-diagnosed	Radiotherapy (30 Gy)	No	No
12	M	59	1	Newly-diagnosed	Without therapy	No	No
13	M	62	12	Newly-diagnosed	Radiotherapy (60 Gy) + temozolomide, adjuvant temozolomide therapy	No	Yes
14	M	71	12	Newly-diagnosed	Radiotherapy (30 Gy) + temozolomide, adjuvant temozolomide therapy	No	Yes

The patients were operated at the Department of Neurosurgery of the University of Ljubljana, University Medical Centre Ljubljana, Slovenia

*M/F* male/female, *IDH1* isocitrate dehydrogenase 1

Tumor cryostat sections of two GBM patients were obtained from the Brain Tumor Bank maintained by the Department of Neuropathology at the Academic Medical Centre (AMC, Amsterdam, The Netherlands) and were used for immunohistochemistry as well as for the detection of the activity of cathepsins B and K using metabolic mapping. Metabolic mapping is not possible in paraffin sections because paraffin embedding inactivates all enzymes. Research was performed on excess tissue that was stored in a coded fashion. Consent for this project was reviewed and waivered, and the project was approved by the Medical Ethics Review Committee of the Academic Medical Center and University of Amsterdam (reference number W14_224 # 14.17.0286). Consent for removal of the tissue and its storage in the tumor bank for research purposes was obtained and documented in the patients’ medical charts. Tissue samples were snap frozen in liquid nitrogen in the operating room and stored at − 80 °C until use. Cryostat sections (7-µm thick) were cut at − 25 °C on an HM560 cryostat (MICROM, Walldorf, Germany), picked up on glass slides, and stored at − 80 °C until use. All staining procedures, including those for controls, were performed on serial sections of each GBM sample.

### Immunohistochemistry

Immunohistochemistry (IHC) was performed on serial cryostat sections (7 µm thick) of two GBM samples and paraffin-embedded sections (5 µm thick) of 14 GBM tumors.

Cryostat sections were air-dried at room temperature for 15 min before staining. Sections were then fixed in acetone (− 20 °C) for 10 min and air-dried afterwards for 15 min. Sections were encircled with a PAP pen (Dako, Glostrup, Denmark), followed by three washing steps of 5 min with 1x phosphate-buffered saline (PBS). The sections were treated with 100% methanol containing 0.5% H_2_O_2_ for 15 min to block endogenous peroxidase activity and to prevent non-specific background staining, followed by three washing steps of 5 min each using PBS. Then, sections were incubated in PBS containing 10% normal goat or rat serum (Dako) and 0.1% bovine serum albumin (BSA; Sigma-Adrich) for 45 min to further reduce nonspecific background staining. After tapping off the serum-containing buffer, sections were incubated overnight at 4 °C with primary antibodies listed in Table 2. After incubation with primary antibodies, sections were washed three times for 5 min in PBS containing 0.1% BSA. Sections incubated with antibodies against Cathepsin K, SMA and SDF-1α were incubated with polyclonal goat-anti-rabbit secondary antibody conjugated with horse-radish peroxidase (HRP) (Dako) in a 1:200 dilution in PBS containing 0.1% BSA for 1 h. Sections incubated with antibodies against cathepsin B and CD133 were incubated with polyclonal rabbit-anti-mouse secondary antibody conjugated with HRP in a 1:200 dilution in PBS containing 0.1% BSA for 1 h. Sections incubated with anti-cathepsin X antibody were incubated with polyclonal rabbit-anti-goat secondary antibody conjugated with HRP (1:200 dilution; Abcam, UK). Incubation with secondary antibodies was followed by three washing steps of 5 min with PBS. Afterwards, sections were incubated with DAB for 10 min, followed by one washing step of 5 min using PBS. Sections were placed in running tap water for 10 min and then in distilled water followed by incubation for 1 min in hematoxylin (Thermo Fisher Scientific, Waltham, MA) to stain nuclei. Sections were again placed in running tap water for 15 min and then in distilled water. All incubation steps were performed at room temperature, except for the overnight incubations with primary antibodies that were performed at 4 °C. Finally, sections were covered with glycerin/gelatin mounting medium (Sigma-Aldrich). Control incubations were performed in the absence of primary antibody.

**Table 2:** Primary antibodies and their dilutions used in immunohistochemical analyses

Primary antibodies	Source and concentration	Dilution cryostat sections	Dilution paraffin sections
Cathepsin B (mouse anti-human)	Faculty of Pharmacy, University of Ljubljana (3E1); 0.918 mg/mL	1:200	1:100
Cathepsin K (rabbit anti-human)	Abcam (ab19027); 0.5 mg/mL	1:200	1:200
Cathepsin X (goat anti-human)	R&D systems (AF934); 0.5 mg/mL	1:200	1:200
CD133 (prominin-1) (mouse anti-human)	Miltenyi Biotech (W6B3C1); 0.1 mg/mL	1:50	1:10
SDF-1α (stromal-derived factor 1α) (rabbit anti-human)	Abcam (ab9797); 0.5 mg/mL	1:200	1:200
SMA (smooth muscle actin) (rabbit anti-human)	Abcam (ab5694); 0.2 mg/mL	1:200	1:200
CD68 (mouse anti-human)	Dako (clone EBM 11); 0.46 mg/mL	/	1:50

Paraffin sections of GBM samples were prepared according to routine procedures of the Institute of Pathology at the University of Ljubljana, Slovenia. Paraffin sections were firstly de-waxed in 100% xylene (3 min) and then rehydrated in 100, 96, 50 and 0% ethanol (in each ethanol dilution for 3 min). Heat-mediated antigen retrieval was achieved with sodium citrate buffer (pH 6.0). The blockage of endogenous peroxidase activity in the tissue was performed by incubation in the presence of 3% H_2_O_2_ in 100% methanol for 30 min at room temperature. To reduce non-specific background staining, sections were incubated in a solution of 10% goat or rabbit normal serum (Dako) in PBS containing 0.1% BSA. After tapping off the serum-containing buffer, sections were incubated overnight at 4 °C with primary antibodies listed in Table 1. Afterwards, sections were incubated with the secondary horse-radish-peroxidase–conjugated antibodies (1:200; Dako) for 1 h at room temperature as described for the staining of cryostat sections. Protein expression was detected using DAB (Dako) or AEC (Vector Laboratories, Burlingame, CA), and haematoxylin was used for counterstaining. The negative-control staining was performed without the addition of primary antibodies. All sections were analyzed by light microscopy and images were taken using a Leica DMLB microscope (Leica, Wetzlar, Germany) and QWin software.

### Determination of cathepsin B and K activity by fluorescence metabolic mapping

**Fig. 1:**
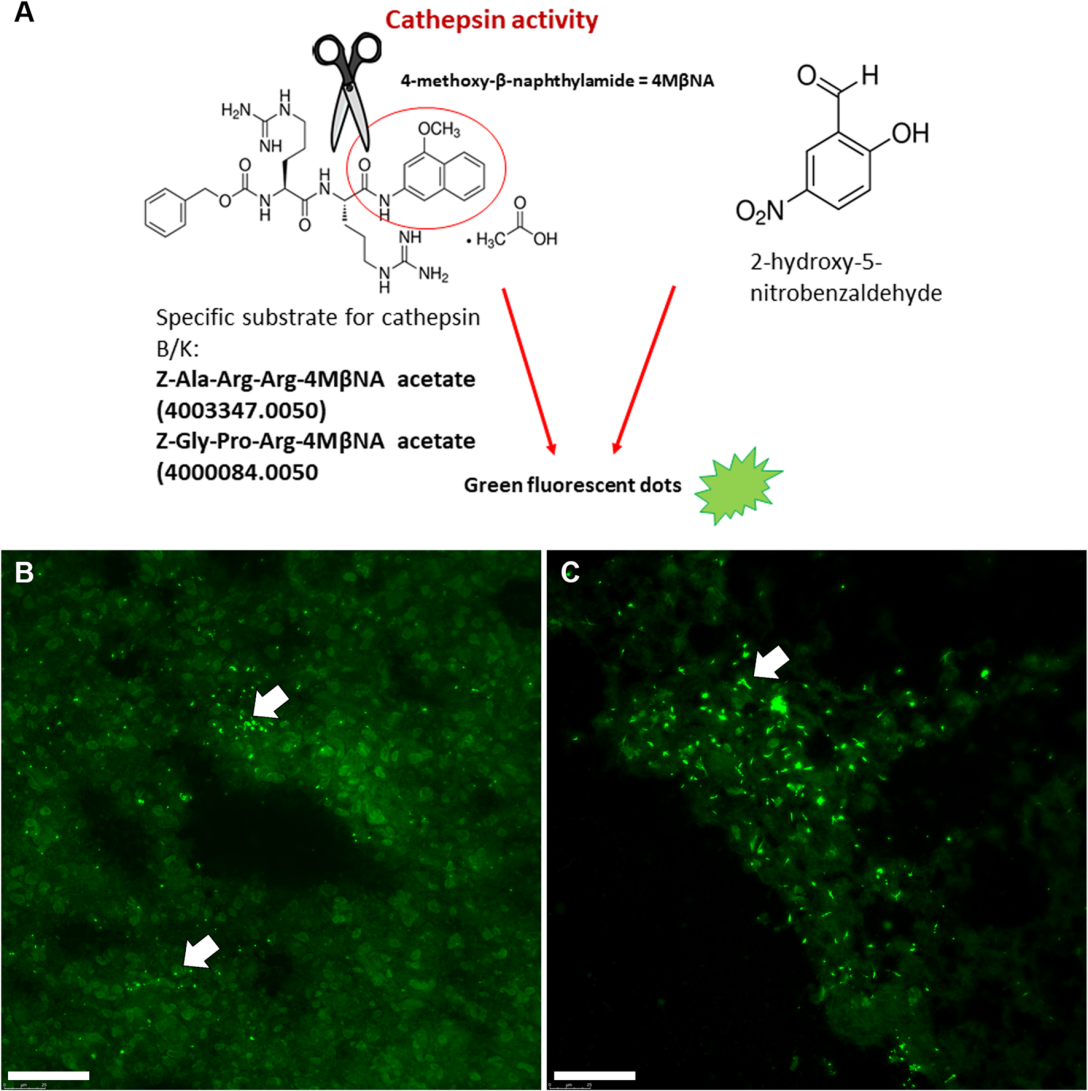
Procedure for localization of cathepsin B and K activity with the use of metabolic mapping and selective fluorogenic substrates. The principle of the monitoring of cathepsin activity in GBM cryostat sections is based on detection of green fluorescent product (dots) obtained after coupling of cleaved 4MβNA with 2-hydroxy-5-nitrobenzaldehyde (**a**). Green fluorescent dots represent cathepsin K activity in GBM sections as indicated by arrows (**b**). Green fluorescent background is due to non-specific binding of 2-hydroxy-5-nitrobenzaldehyde to NH_2_ groups of proteins. After 50–60 min, recrystallization of the final reaction product starts to occur leading to the formation of needle-like crystals (**c**; indicated by arrow). Thus, the images need to be captured before recrystallization starts to occur. Scale bar = 50 µm. (Color figure online)

The activities of cathepsins B and K in GBM serial sections were detected using fluorescence metabolic mapping as described by Van Noorden et al. (1987) and Hazen et al. (2000). This method is based on the detection of protease activity by coupling 2-hydroxy-5-nitrobenzaldehyde (NSA) with cleaved 4-methoxy-β-naphthylamide (4MβNA), resulting in green fluorescent coupling products. NSA binds also non-specifically to NH_2_ groups in proteins, giving rise to a green fluorescent background (Fig. 1). By imaging sections while they are incubated in medium containing specific substrate and NSA, the formation of green fluorescent final reaction product can be followed in time. Unfixed GBM cryostat sections were air-dried for 10 min and incubated with a drop of incubation medium containing dithiotreitol (final concentration 1 mM; Amresco), EDTA (final concentration 1.3 mM; Sigma), l-cysteine (final concentration 2.7 mM; Merck), 2-hydroxy-5-nitrobenzaldehyde (final concentration 1 mM; Sigma-Aldrich) and specific substrate of the cathepsins (final concentration 1 mg/mL), all dissolved in 100 mM phosphate buffer (pH 6.0). For determination of cathepsin B and K activity, the substrates Z-Ala-Arg-Arg-4MβNA acetate and Z-Gly-Pro-Arg-4MβNA acetate (both Bachem, Bubendorf, Switzerland) were used, respectively. After covering the medium on the top of the section by a coverslip, the incubations were performed at a constant room temperature on the stage of a fluorescence microscope Leica DMF6 B for time intervals up to 90 min. Images were taken while the tissue sections were incubated for cathepsin activity every 30 s in a period from 1 to 90 min using a FITC filter (excitation filter 440–500 nm; emission filter 515 nm). The images were taken using Leica Application Suite X software and a Leica DFC 9000 GT camera. All images were taken under the same settings such as exposure time and laser power. The control incubations were carried out in the presence of the cathepsin inhibitor leupeptin (final concentration 5 µM; Sigma) and in the absence of the specific substrate.

## Results

### Cysteine cathepsins B, X and K are expressed in glioblastoma cells and stromal cells

**Fig. 2:**
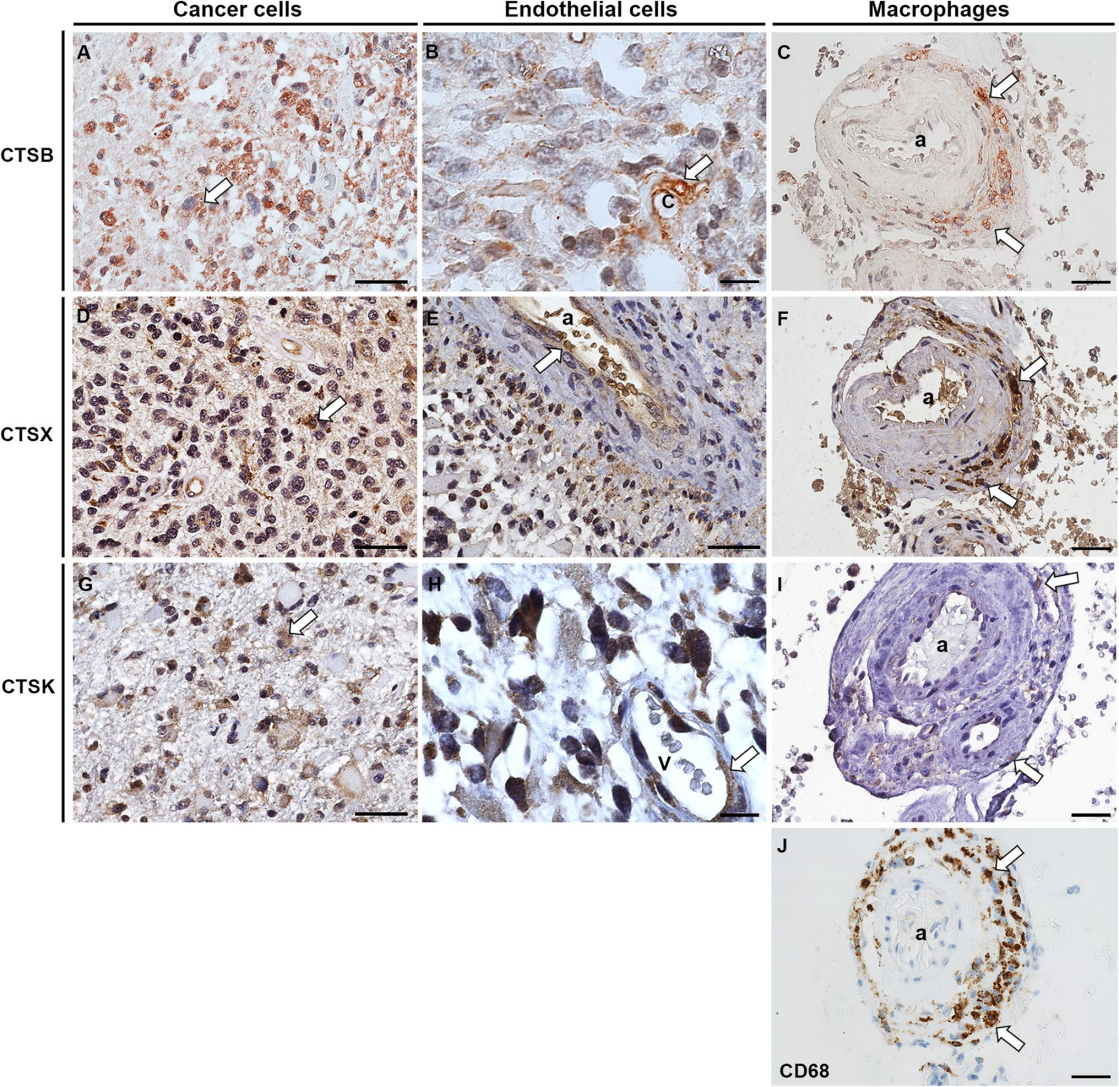
Immunohistochemical staining of cathepsins (CTS) B, X and K in paraffin-embedded sections of human GBM. Protein expression of cathepsins B, X and K was present in GBM cells (**a, d, g**; indicated by arrows), endothelial cells of blood vessels (**b, e, h**; indicated by arrows) and in CD68-positive macrophages (**c, f, i, j**; indicated by arrows). CD68 expression was detected in the wall of arterioles (**j**; indicated by arrows) and the staining of CD68 overlapped with the staining of cathepsins B and X (**c, f**; indicated by arrows), and to a lesser degree with cathepsin K (**i**; indicated by arrows) in serial GBM sections. Imunohistochemical labelling of cathepsin B was detected using AEC substrate (red color) and that of other antigens using DAB substrate (brown color). Cell nuclei were counterstained using haematoxylin (blue/purple). *a* arteriole; *c* capillary; *v* venule. **a, d, g, e**: scale bar = 30 µm, x400 magnification; **b, h**: scale bar = 10 µm; ×1000 magnification; **c, f, i, j**: scale bar = 20 µm; ×400 magnification. (Color figure online)

We examined expression of cathepsins B, X and K in GBM tumor samples of 16 patients by performing IHC on GBM sections. The intensity of immunostaining and the proportion of stained cells were heterogeneous within tumor samples and varied between individual GBM tumors. Cathepsins B was expressed in all 16 GBM tumors in GBM cells (Fig. 2a), endothelial cells (Fig. 2b) and in macrophages/microglia cells (Fig. 2c, j). Similarly to cathepsin B, cathepsin X was expressed in all GBM tumors in GBM cells (Fig. 2d), endothelial cells (Fig. 2e) and in macrophages/microglia cells (Fig. 2f, j). Cathepsin K was expressed in GBM cells (Fig. 2g) and endothelial cells (Fig. 2h). In contrast to cathepsins B and X, cathepsin K protein expression was not found in all GBM samples and its immunostaining signal was weaker as compared to that of cathepsins B and X (Fig. 2g). IHC-stained sections exhibited ubiquitous and granular staining of cathepsins B and X (Fig. 2a, d). The presence of cathepsins in macrophages was demonstrated by comparing immunostaining patterns of cathepsins and the macrophage marker CD68 in consecutive sections of the same GBM sample (Fig. 2c, f, i, j). There was hardly any expression of cathepsin K in CD68-positive cells in overlapping regions in the sections (Fig. 2i, j). In addition, immunostaining of all three cathepsins was detected in normal brain tissue around the tumor, and in microglia cells (data not shown).

### Peri-arteriolar GSC niches in GBM samples

**Fig. 3:**
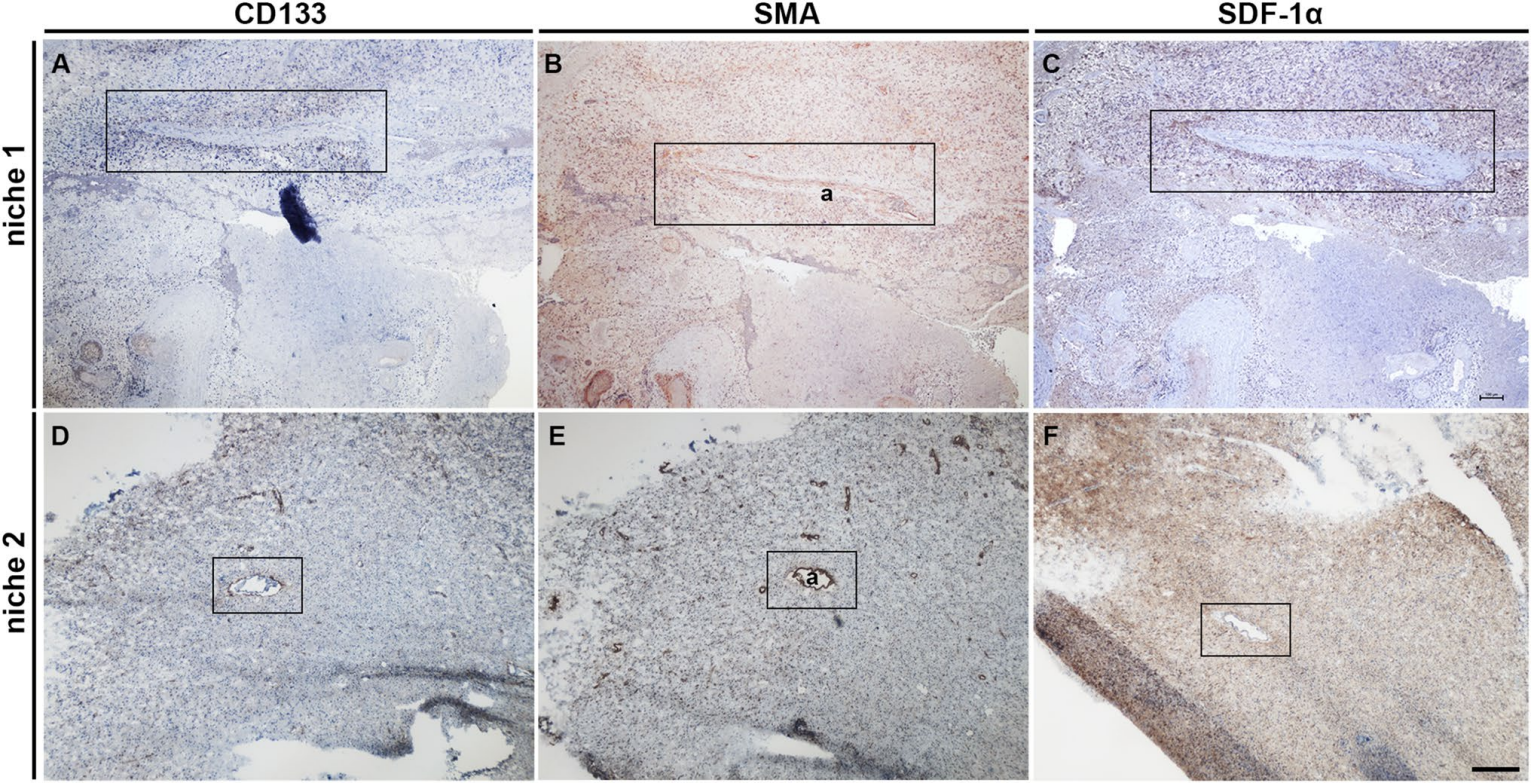
Low-magnification images of peri-arteriolar GSC niches in paraffin-embedded sections (niche 1) and cryostat sections (niche 2) of GBM. Serial sections of GBM were screened for GSC niche regions after IHC staining of the stem cell marker CD133 (**a, d**) at a low magnification (×4). GSC niches were identified by immunolabelling of SMA for smooth muscle cells of arterioles (**b, e**) and the cytokine and niche factor SDF-1α (**c, f**). Niche 1 and niche 2 were found in different GBM patient samples. Immunohistochemical labelling of SMA in paraffin-embedded sections was performed with AEC as chromogen (**b**; red color) and of SMA in cryostat sections and other antigens with DAB as chromogen (**a, c**-**f**; brown color). Cell nuclei were counterstained using haematoxylin (blue/purple). Niches are marked by rectangles. *a* arteriole. Scale bar = 200 µm. (Color figure online)

**Fig. 4:**
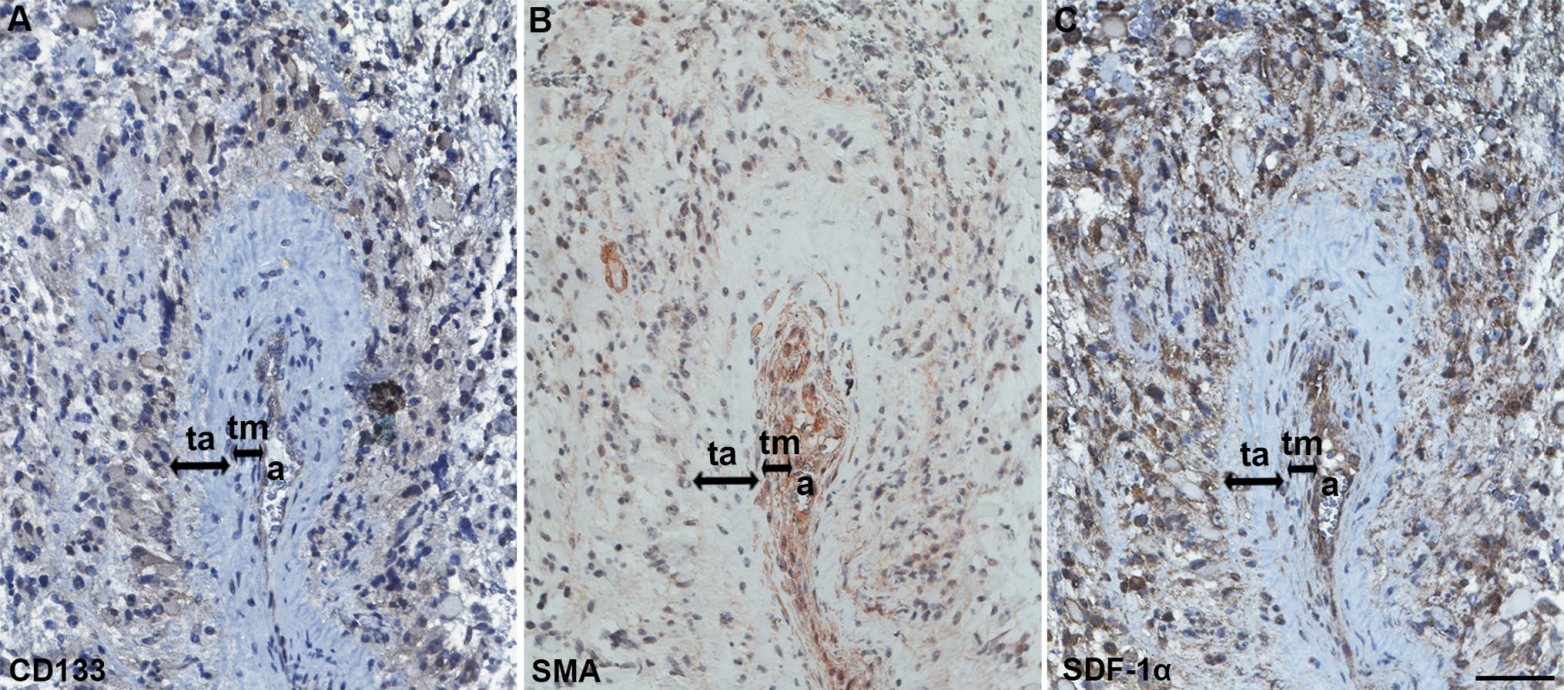
High-magnification images of GSC niche markers CD133, SMA and SDF-1α in peri-arteriolar GSC niches in serial paraffin-embedded GBM sections. CD133-positive cells were adjacent to the tunica adventitia of an arteriole (**a**). SMA-positive smooth muscle cells were present in the tunica media of the arteriole (**b**). SDF-1α staining was present in cells adjacent to the tunica adventitia of the arteriole and in endothelial cells of the arteriole (**c**). Immunohistochemical labelling of SMA was performed with AEC as chromogen (red color) and of the other antigens with DAB as chromogen (brown color). Cell nuclei were counterstained using haematoxylin (blue/purple). *a* lumen of arteriole, *ta* tunica adventitia, *tm* tunica media. Scale bar = 50 µm. (Color figure online)

We identified peri-arteriolar GSC niches in sections of GBM samples based on immunohistochemical localization of cancer stem cell marker CD133, smooth muscle actin (SMA) as smooth muscle cell marker and cytokine SDF-1α, which have been shown to be associated with GSC niches (Hira et al. 2015, 2018b) (Fig. 3). Briefly, CD133 is the most widely-used GSC marker (Singh et al. 2004; Zeppernick et al. 2008; Ardebili et al. 2011; Podergajs et al. 2016). SMA is localized in smooth muscle cells in the tunica media of arteriolar and venular walls and SDF-1α, a chemotactic cytokine, is produced by endothelial cells and is involved in the retention and maintenance of GSCs within their niches (Plaks et al. 2015; Hira et al. 2017a). We observed that stem cell marker CD133 was expressed in 15 GBM samples out of 16. In these 15 GBMs, CD133 immunostaining was present only in a few areas of the sections. In 9 out of these 15 CD133-positive samples, staining was observed mostly in cells adjacent to the tunica adventitia of arterioles with a thick layer of SMA-positive smooth muscle cells in the tunica media (Fig. 4a, b). Strong SDF-1α staining was found in sections of all GBM samples in various regions of GBM tissue, including endothelial cells and cells adjacent to the tunica adventitia of arterioles, that were positive for CD133 and SMA (Fig. 4c). In addition, the immunostaining signal of SMA was dependent on the number of arterioles in GBM tumors. When the number of arterioles in a GBM sample was high, the imunostaining signal of SMA was observed more frequently within the GBM tissue sections.

### Expression of cathepsins B, X and K around arterioles

**Fig. 5:**
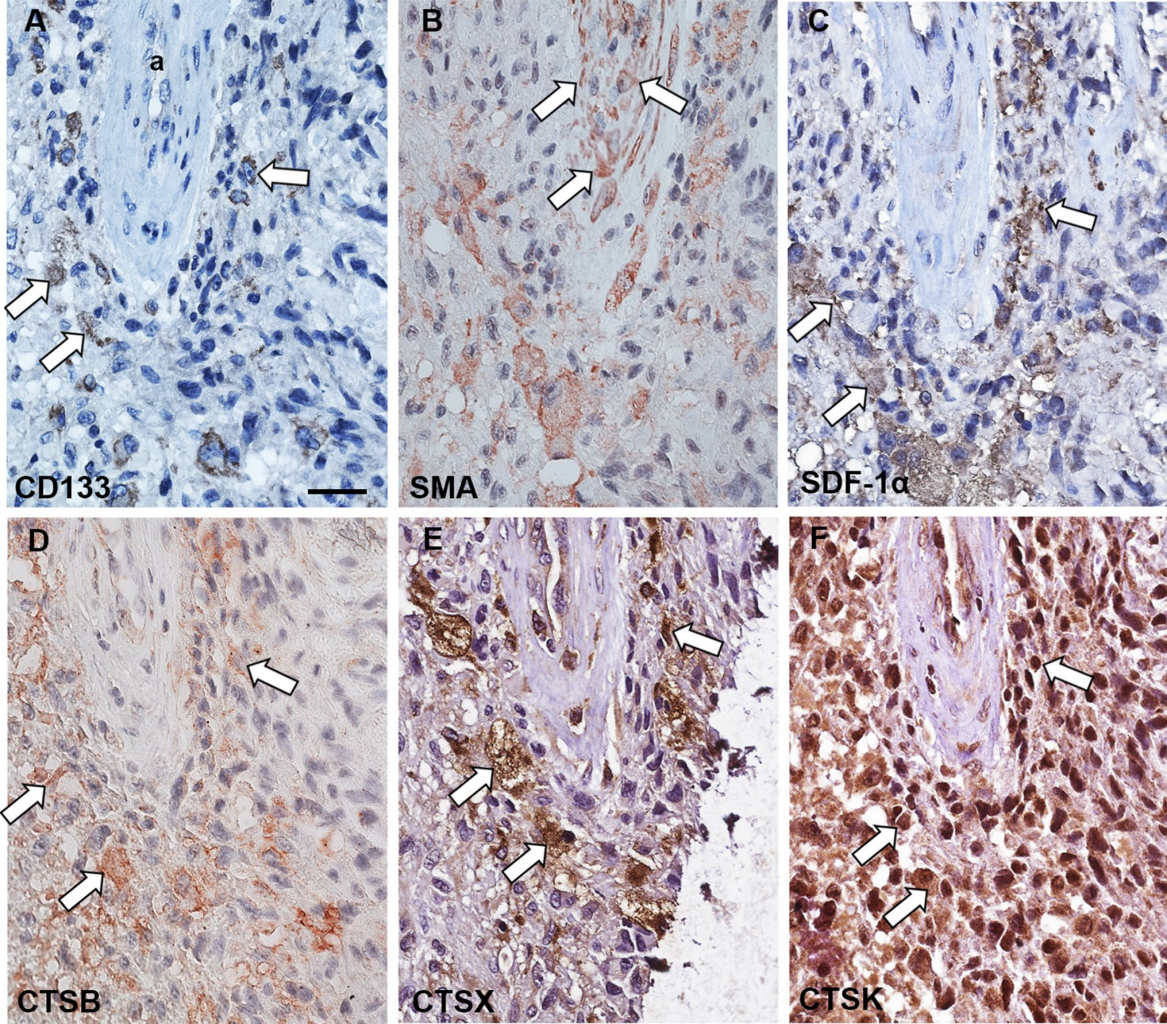
Serial paraffin-embedded GBM sections immunohistochemically stained for cathepsins B, X and K in peri-arteriolar GSC niches. CD133-positive cells (**a**; indicated by white arrows) were localized adjacent to the tunica adventitia of SMA-positive arterioles (**b**; SMA-positive cells in the tunica media of the arteriole are indicated by white arrows) where niche factor SDF-1α was expressed in high abundance (**c**; indicated by white arrows). Cathepsin B (**d**), X (**e**) and K (**f**) were expressed in GSC niches as indicated by white arrows and their staining overlapped with the staining of CD133 and SDF-1α (**a, c**). Niche 1 of Fig. 3 is presented. Immunohistochemical labelling of cathepsin B and SMA was performed with AEC as chromogen (red color) and of the other antigens with DAB as chromogen (brown color). Cell nuclei were counterstained using haematoxylin (blue/purple). *a* arteriole. Scale bar = 20 µm. (Color figure online)

**Fig. 6:**
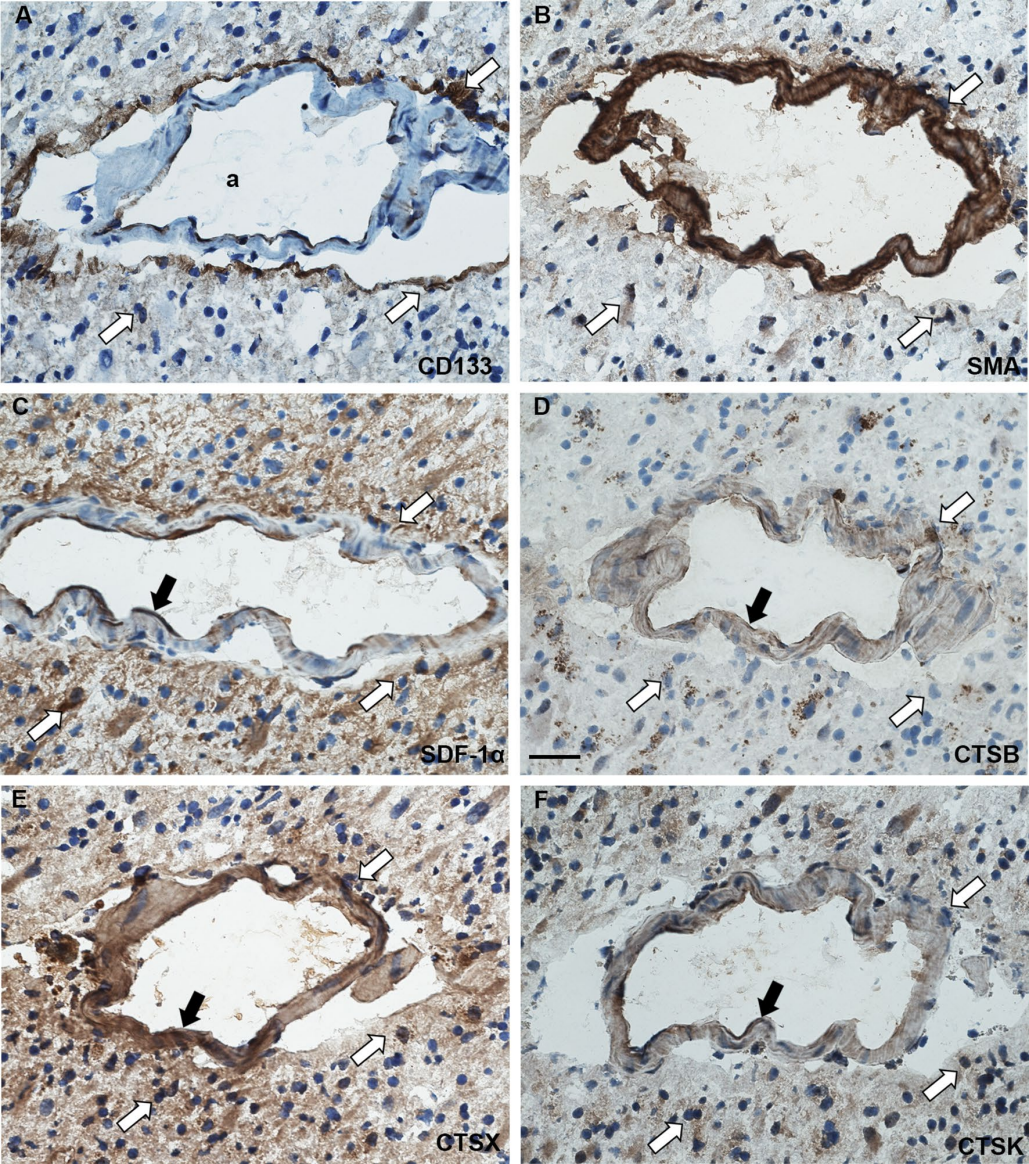
Serial cryostat GBM sections immunohistochemically stained for cathepsins B, X and K in peri-arteriolar GSC niches. CD133 was expressed in distinct GSCs localized around arterioles (**a**; indicated by white arrows). SMA expression was found in the smooth muscle cells of the arteriolar wall and GSCs around arterioles as indicated by white arrows (**b**). SDF-1α expression was associated with CD133-positive GSCs (indicated by white arrows) and endothelial cells of arterioles as indicated by black arrow (**c**). Cathepsin B expression was present in GSCs/GBM cells close to arterioles as indicated by white arrows and also at a distance from the niche, where focal and diffuse staining was observed, and in endothelial cells as indicated by black arrow (**d**). Cathepsin X was most strongly expressed in GSCs as indicated by white arrows and in endothelial cells in the niche as indicated by black arrow (**e**). Cathepsin K was expressed in GSCs as indicated by white arrows and in endothelial cells as indicated by black arrow (**f**). Niche 2 of Fig. 3 is presented. Immunohistochemical labelling of proteins was performed with DAB as chromogen (brown color). *a* arteriole. Scale bar = 20 µm. (Color figure online)

**Fig. 7:**
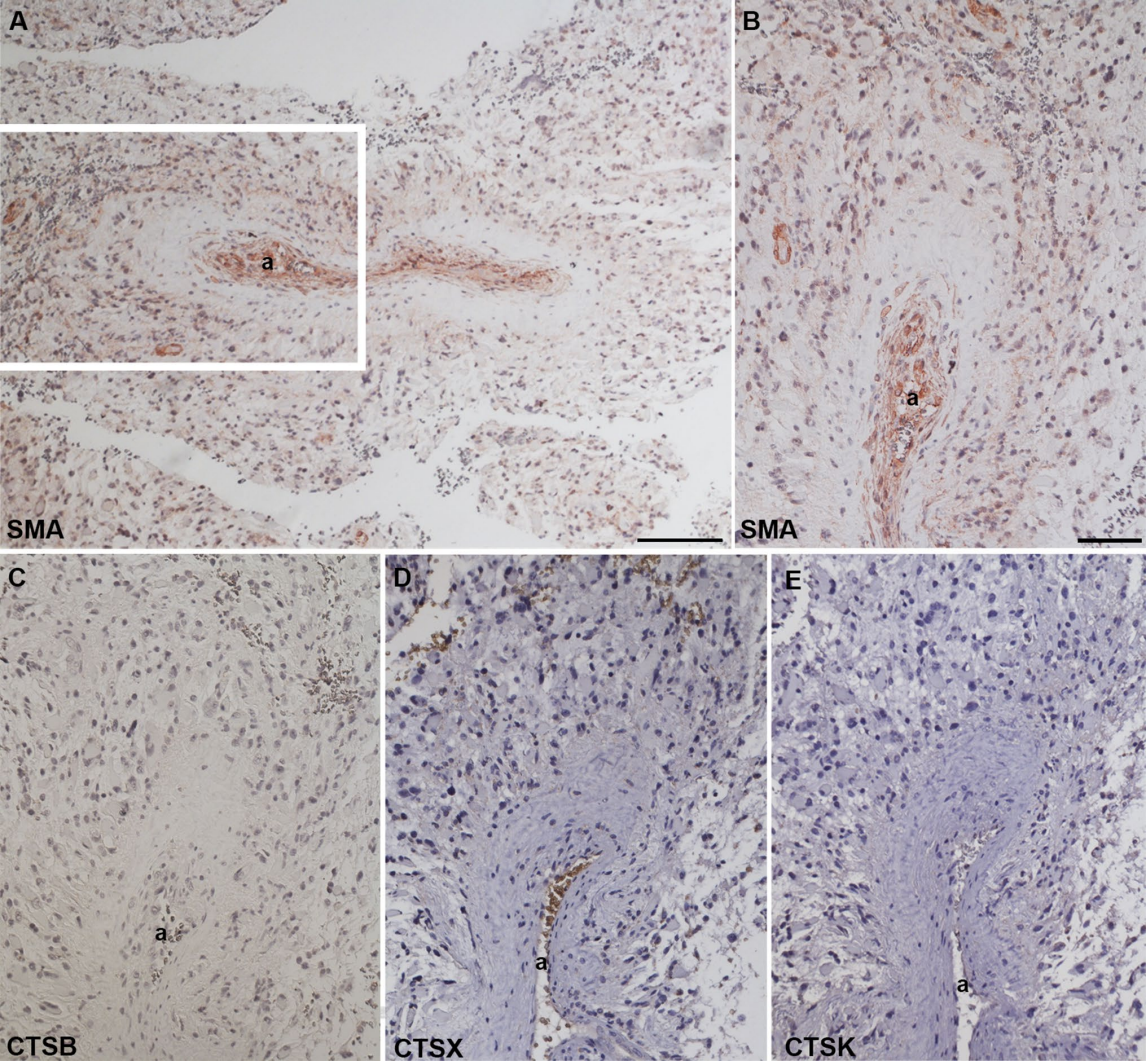
Arteriole in serial paraffin-embedded GBM sections without cathepsin expression. SMA-positive smooth muscle cells were present in the tunica media of the arteriole (**a, b**). The region around the arteriole as shown in **a** was enlarged. Cathepsin B, X and K expression was absent around the arteriole (**c**–**e**). Non-specific staining of erythrocytes was detected in the lumen of the arteriole (**d**). Immunohistochemical labelling was detected using DAB as chromogen (**a**–**c**) or AEC as chromogen (**d, e**). Cell nuclei were counterstained using haematoxylin (blue/purple). *a* arteriole. **a** scale bar = 100 µm; **b**–**e**: scale bar = 50 µm. (Color figure online)

We observed that cathepsins B, X and K were localized around the tunica adventitia of arterioles (Figs. 5, 6), cathepsin B in 6 out of 166 arterioles, cathepsin X in 49 out of 166 and cathepsin K in 45 out of 166 arterioles. The localization of cathepsin K was distinctly present in specific cells, whereas cathepsin B and X were present in most cancer cells surrounding the arterioles. Moreover, cathepsins B and X were found in macrophages around arterioles. Overall, cathepsin B and X expression was detected in high abundancy throughout the GBM sections, whereas cathepsin K expression was more restricted to arteriolar areas in GBM tissue sections. Figure 7 shows an arteriole without cathepsin expression.

### Cathepsins B, X and K are localized in peri-arteriolar GSC niches

**Fig. 8:**
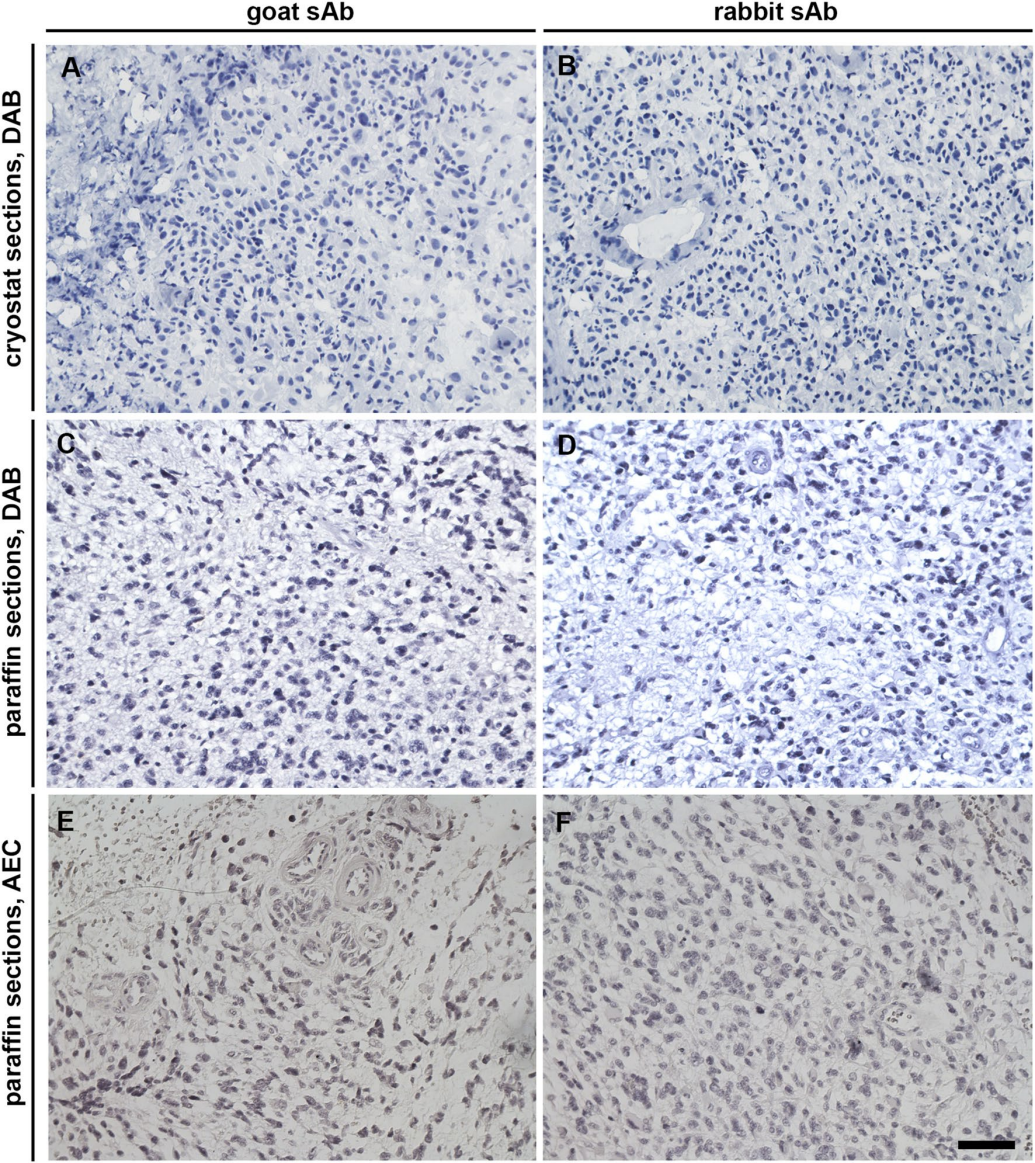
Control incubations for IHC of cryostat and paraffin-embedded GBM sections. Control incubations were performed in the absence of primary antibodies and with goat (**a, c, e**) or rabbit (**b, d, f**) secondary horse-radish-peroxidase–conjugated antibodies (sAb) in the final dilution of 1:200. Immunohistochemical labelling was detected using DAB as chromogen (**a**–**d**) or AEC as chromogen (**e, f**). Cell nuclei were counterstained using haematoxylin (blue/purple). Scale bar = 50 µm. (Color figure online)

Cathepsins B, X and K were expressed in the regions of peri-arteriolar GSC niches, which were positive for niche markers CD133, SMA and SDF-1α, in paraffin-embedded (Fig. 5) and cryostat serial GBM sections (Fig. 6). Cathepsins were present in CD133-positive cells adjacent to the tunica adventitia of arterioles (Fig. 6a, d–f) as well as in SDF-1α-positive endothelial cells of SMA-positive arterioles (Fig. 6c–f). Taken together, cathepsin B, X and K expression was found in peri-arteriolar GSC niches in 9 out of 16 GBM samples, although not all peri-arteriolar GSC niches in these 9 GBM samples were positive for all three cathepsins. Expression of cathepsin B, X and K was not mutually exclusive. In some peri-arteriolar GSC niche regions only one or two cathepsins were expressed, whereas in others all three cathepsins were expressed. We tested the correlation between the survival of GBM patients and the presence of the niches in GBM samples and did not find any correlation (data not shown). However, due to the limited number of GBM samples it is hard to come to conclusions. The negative control staining was performed without the addition of primary antibodies and is shown in Fig. 8.

### Cathepsin B is proteolytically active in peri-arteriolar GSC niches

**Fig. 9:**
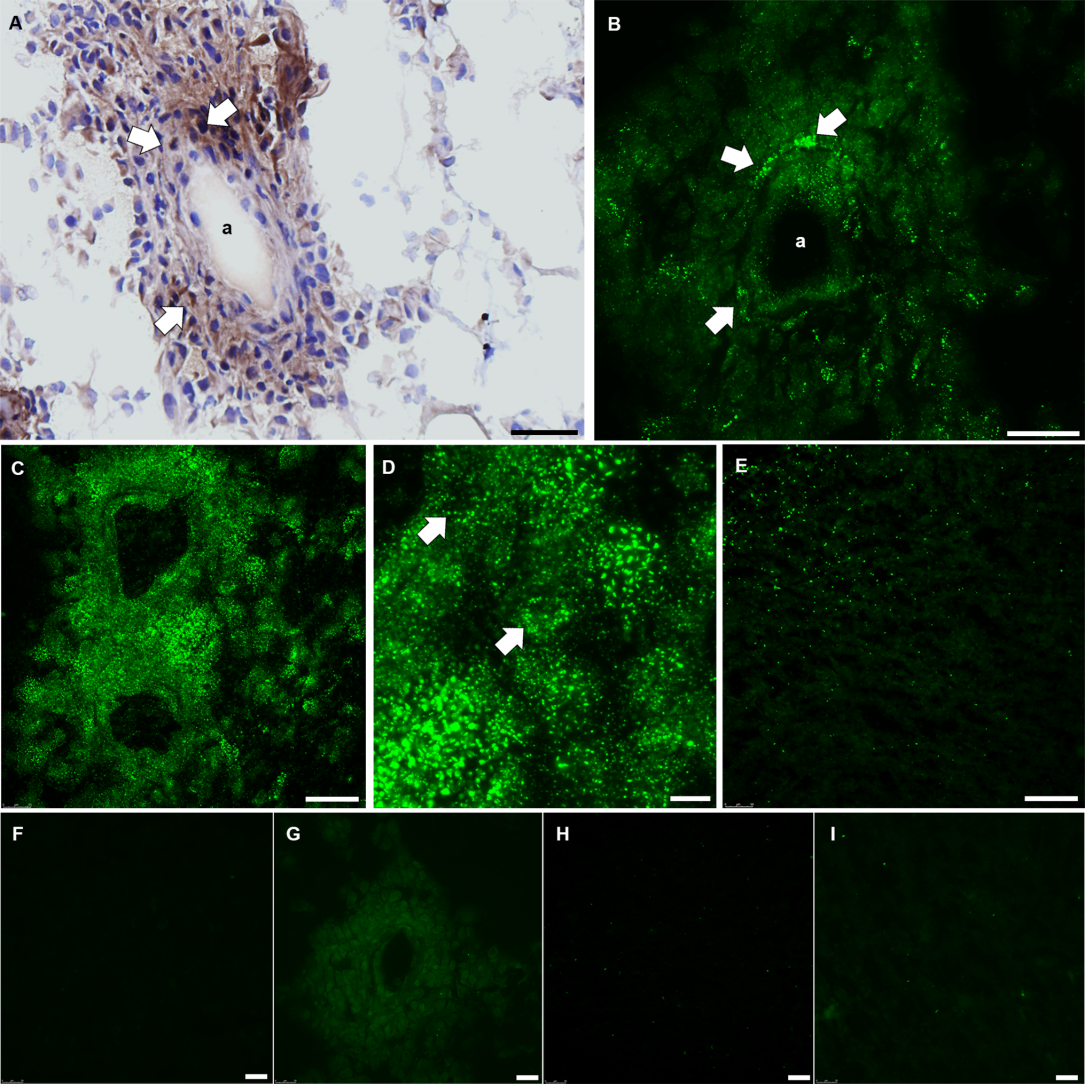
Activity of cathepsins B and K in GBM cryostat sections. Cathepsin B protein expression around an arteriole in a GBM cryostat section (**a**). Green fluorescent dots, representing activity of cathepsin B in the same GSC niche as cathepsin B protein expression in **a** (**b**). Cathepsin B activity was detected throughout the GBM tissue (**c**). Higher magnification of a GBM tissue section showing cathepsin B activity (**d**). Arrows indicate cathepsin B activity (granular green fluorescent coupling product) in the cells. Cathepsin K activity was detected in some regions of GBM tissue, but not in GSC niches (**e**). The incubations with protease inhibitor leupeptin and cathepsin B substrate (**f**), incubations in the absence of cathespin B substrate (**g**), incubations with leupeptin and cathepsin K substrate (**h**), and incubations in the absence of cathespin K substrate (**i**) did not produce granular green fluorescent reaction product. *a* arteriole. **a**, **d**, **e**: scale bar = 50 μm; **b** scale bar = 10 μm; **c** scale bar = 100 μm; **f**–**i**: scale bar = 25 μm. (Color figure online)

In addition to protein expression of cathepsins, we examined the proteolytic activity of cathepsins B and K in serial cryostat sections of the two frozen GBM samples using fluorogenic metabolic mapping. Both GBM samples gave similar results with respect to expression of all three cathepsins in tumor sections. Both GBMs exhibited high expression of cathepsin B an X and lower expression of cathepsin K. Also, the localization of all three cathepsins was similar in these two GBM tissues. Cathepsin B and X were expressed in GSCs around arterioles but also in other GBM cells, macrophages and endothelial cells in other regions of the tissues. On the other hand, cathepsin K expression was more restricted to the areas around arterioles in GSCs and endothelial cells. Fluorogenic metabolic mapping enabled us to localize and image proteolytically-active proteases in specific regions of the tumors such as GSC niches. Similar to the protein expression of cathepsin B (Fig. 9a), high cathepsin B activity was detected in GBM cryostat sections of the two frozen GBM samples (Fig. 9b–d). The green dots, representing final reaction product, started to appear after 5 min incubation in the substrate- and NSA-containing medium. After 45 min, green fluorescent dots were observed in cells in the tissue sections, localized close to the nuclei (probably lysosomes) and in the peripheral parts of the cells (Fig. 9b, d). After 50 min, recrystallization of the final fluorescent reaction product started to occur, resulting in the formation of needle-like crystals. We detected the activity of cathepsin B in cells within peri-arteriolar GSC niches in both GBM samples (Fig. 9b). When we tested the activity of cathepsin K, only few green fluorescence dots of the final reaction product appeared after 20–30 min of incubation. We observed that cathepsin K was hardly proteolytically active and only in a limited number of regions in sections of both GBM samples (Fig. 9e). After 50–60 min, recrystallization of the final fluorescent reaction product started, resulting in the formation of needle-like crystals. We did not detect cathepsin K activity in GSC niches in both GBM samples. Incubations with an inhibitor of serine and cysteine proteases, leupeptin, and the incubations in the absence of the specific substrate, did not produce green fluorescent reaction product (Fig. 9f–i). We were not able to demonstrate the activity of cathepsin X because a selective fluorogenic substrate is not available.

## Discussion

In the present study, we analyzed cellular localization of proteases cathepsins B, X and K in the peri-arteriolar GSC niches by IHC. Their protein expression patterns gives an indication whether cathepsins B and X are also functionally involved in modulating GSC trafficking and GSC maintenance in the niches. GSC niches are regions in GBM tumors, where GSCs are surrounded by specific ECM proteins and stromal cells, predominantly endothelial cells and pericytes of vessels, macrophages, stromal fibroblasts and possibly other brain stromal components (Calabrese et al. 2007; Breznik et al. 2017b; Roos et al. 2017; Hira et al. 2018a). Juxtacrine signaling between endothelial cells and GBM cells (Calabrese et al. 2007) is crucial to maintain the stem cell phenotype of GSCs (Zhu et al. 2011; Schiffer et al. 2014; Roos et al. 2017). Recently, the different types of cancer stem cell niches, such as peri-vascular, peri-hypoxic, peri-immune, ECM and peri-arteriolar niches were integrated into one concept, the hypoxic peri-arteriolar GSC niche (Hira et al. 2018a), which mimics the HSC niche in bone marrow (Hira et al. 2018b). In the same study, GSC niches were found around 2% of the arterioles and 0% of venules and capillaries.

Cathepsin K, the first cathepsin that was found to be associated with the niche (Hira et al. 2015, 2017a), was localized in GBM tumors in GBM cells, endothelial cells, and in GSC niches adjacent to the tunica adventitia of arterioles. We also detected very limited cathepsin K activity in GBM sections with the use of a highly sensitive fluorogenic method (Van Noorden et al. 1987; Hazen et al. 2000), but this activity was not associated with arteriolar walls or GSC niches. In a previous study, we found high levels of cathepsin K pro-form but not its active form. In the same study, the activity of cathepsin K was not found in GBM sections whereas osteoclasts in mouse bone cryostat sections as a positive control appeared to be strongly positive (Verbovšek et al. 2014). This indicates tight regulation of cathepsin K activity by cathepsin K-activating proteases and the extracellular matrix/ microenvironment (pH, redox potential) (Novinec and Lenarčič 2013).

Cathepsin B protein expression was found to be associated with GBM cells, macrophages/microglia cells and endothelial cells, confirming our previous studies on its distribution pattern in glioma tissue sections (Strojnik et al. 2005). Moreover, we found cathepsin B to be present in GSCs and macrophages/microglia cells around arterioles. Metabolic mapping revealed that cathepsin B was highly active in the GSC niche areas. This may be due to high SDF-1α activity that is mediating the paracrine interactions between endothelial cells and GBM cells, as was demonstrated in an in vitro model (Kenig et al. 2010). The authors showed that endothelial cells affect GBM cells, resulting in enhanced invasion of GBM cells towards the endothelial cells as well as increased endothelial cells proliferation via a mechanism involving enhanced cathepsin B activity.

Cathepsin X staining was abundant in all 16 GBM samples. Cathepsin X protein was present in macrophages/microglia and endothelial cells and was also found in a layer adjacent to the tunica adventitia of arterioles. In contrast to other cathepsins, cathepsin X is involved in cancer cell adhesion, migration and invasion via interactions with integrin receptors (Kos et al. 2009, 2015), as was also shown in an experimental mouse model by Akkari et al. (2014). These studies showed that several tumor-promoting functions of cathepsin X were not dependent on its catalytic activity but were rather mediated via the Arg–Gly–Asp (RGD) motif in the prodomain of the enzyme, which regulates interactions with integrins and the ECM. As integrins are enriched in GSCs and are participating in GSC self-renewal and therapy resistance (Lathia et al. 2010; Seguin et al. 2015), cathepsin X modulation of integrins may also affect GSC fate in their niches. Taken together, the IHC staining patterns revealed that cathepsin B and X proteins are abundantly expressed in GBM tumors, whereas cathepsin K expression is more limited to peri-arteriolar regions, but none of the three cathepsins was exclusively expressed in GSC niches. Therefore, we cannot rule out that the cathepsins have specific functional roles in GSC niches. Any association between clinical data and staining differences was not possible due to relative low number of tumors (n = 16) investigated and the intratumoral heterogeneity.

With respect to the cysteine cathepsins’ functionality, Staudt et al. (2012) showed that cathepsins B, X and K are structurally and functionally diverse, but they share the ability to proteolytically cleave the chemotactic cytokines, such as SDF-1α, although at different sites. As discussed above, this chemokine is present in high abundance in the GSC niches and we have previously demonstrated (Hira et al. 2017a) that CXCR4/CXCR7-positive GSCs migrate towards a gradient of SDF-1α that retains GSCs in peri-arteriolar niches. Moreover, we have clearly demonstrated that cathepsin K specifically hydrolyses up to 20 amino acids of the N-terminus of SDF-1α, that contains its active site and thus inhibits chemotactic activity towards CXCR4/CXCR7-positive GBM cells and GSCs in vitro. In addition, the chemoattraction of SDF-1α towards GSCs was inhibited in the presence of the CXCR4 inhibitor plerixafor. On the other hand, Staudt et al. (2012) claimed that only cathepsin B carboxypeptidase activity impairs the SDF-1α chemotactic activity. This cytokine also retains HSCs in their niches in bone marrow, and cleavage of SDF-1α by cathepsin B caused the reduction in chemotaxis of HSCs. In addition, the same authors proposed that cathepsin X proform is directly involved in stem cell trafficking within the niche via modulation of cell adhesion proteins. For example, pro-cathepsin X treatment with dithiothreitol, which unfolds it, but does not lead to full activity of cathepsin X, significantly reduced HSC adhesion to osteoblasts (Staudt et al. 2012). These observations argue for a role of the accessible cathepsin X prodomain in GSC binding in their niches. Altogether, since cathepsins B, X and K are all present in GSC niches, there is a possibility that all three cathepsins cooperate in the regulation of SDF-1α chemotactic activity in vivo.

Taken together, we localized the cysteine cathepsins B, X and K in association with SMA-positive arterioles and CD133 as cancer stem cell marker and the GSC niche marker SDF-1α. Moreover, we detected high levels of cathepsin B activity and low levels of cathepsin K activity in the GBM sections. The localization of cathepsin K is in agreement with a functional role in GSC niches in peri-arteriolar regions. The presence of cathepsins B and X was abundant in the GBM samples and its localization was not specifically associated with the peri-arteriolar GSC niches. In conclusion, as it still poorly understood which molecular programs and proteins exert specific functions in GSC niches, identification of proteins and signaling pathways within GSC niches may have therapeutic value, because niche-residing GSCs are therapy resistant as they are slowly dividing cells. Thus, targeting and/or disintegrating niches may reduce affinity of GSCs to niches and may induce differentiation of GSCs into proliferative GBM cells, subsequently increasing their sensitivity to anti-cancer therapy (Hira et al. 2017a).
